# On Criminal Insanity

**Published:** 1848-01-01

**Authors:** 


ART. IV.—(1.) *Clinical Facts and Reflections; also, Remarks on the Impunity of Murder in some cases of Presumed Insanity.* By THOMAS MAYO, M.D. London, 1847.

(2.) *The Consciousness of Right and Wrong; a Just Test of the Plea of Partial Insanity in Criminal Cases,* (*reprinted from No.* 172 *of the Edinburgh Medical and Surgical Journal.*) By C. LOCKHART ROBERTSON, M.D. Edinburgh, 1847.

WHILE all agree that the plea of insanity may, and that it in many instances does, absolve from the consequences of criminal acts, the greatest diversity of opinion exists as to the responsibility or irresponsibility to be attached to individual acts, the result of the individual varieties of mental disease.

Hence the law, assuming it as an axiom that insanity may or may not render its victim irresponsible, has endeavoured to frame a test whereby the measure of responsibility may be so meted that the ends of justice in the prevention and punishment of crime, as the dictates of humanity in the acquittal of those by disease deprived of their responsibility, be alike attained.

This test, according to the law of England, is the presence or absence, at the time of committing the act, in one of diseased mind, of the consciousness of right and wrong. "Before a plea of insanity," say the fifteen judges,* "should be allowed, undoubted evidence ought to be adduced that the accused was of diseased mind, and that at the time he committed the act he was not conscious of right and wrong." And again, "that nothing could justify a wrong act, except it was clearly proved that the party did not know right from wrong."

When in criminal cases the plea of insanity is set up, the usual practice is for the jury, empannelled to try the guilt of the accused, likewise to decide on the evidence adduced how far mental disease has rendered the party irresponsible.

To the solving of this difficulty, our juries are, as at present constituted, totally unfit. Respectable men, but ignorant alike of the constitution of the human mind in health and of its varied symptoms in disease, it is strange they have ventured, and been so long suffered, and that too when the life of a human being hangs in the scale, to decide this important question.

No wonder there is no unanimity of principle in their verdicts! And well may Dr. Mayo remark, that "the philosopher cannot fail to observe, that the views both of judges and advocates themselves are insensibly lowered down to the dimensions of the very incompetent body in whom the decision ultimately rests." (p. 179.)

And yet, on the majesty of England's law, and on the value of the test furnished by it, has all the slur been cast, when jurors, alike ignorant of philosophy and of medicine, have failed rightly to administer the same; when even the very test has ofttimes been falsely applied to the case in question by physicians ignorant of mental disease.

The accurate diagnosis of bodily disorder requires a knowledge alike of healthy and morbid structure, and he who without this knowledge would attempt to decide upon the presence of and to treat disease is but an empiric.

And so, but in a far higher degree, does the correct discrimination of mental disease demand a perfect knowledge of the healthy action of the mind—of mental philosophy and of mental pathology in its varied phases of moral and intellectual disorders,—and the physician who without these requisites would attempt to apply to individual cases of insanity the legal test of responsibility and irresponsibility, is, whatever his other qualifications may be, acting in this instance the part of an empiric, and dishonestly undertaking a task he must feel himself unfit to grapple with.

Taking, then, these circumstances into consideration—the frequent incapacity of the medical witnesses to judge of the state of mind of the

* Answers to the questions proposed to them by the House of Lords, June, 1843.

accused, the constant unfitness of the jury for the forming of a just verdict,—*we believe it would be better in all instances to try the guilt by the jury, and then, in any suggested case of insanity, to have a separate inquiry as to the state of the criminal's mind and capacity, conducted by physicians conversant with mental disease.*

Before entering on the examination with what accuracy the legal test—viz., the consciousness of right and wrong—decides in the many varieties of mental disease on the responsibility or irresponsibility present, it is incumbent on us to ascertain whether in the healthy mind (from which all our conclusions as to the diseased mind ought to be deduced) there exists the consciousness of right and wrong, or, as philosophers term it, the moral principle; and whether this moral principle, or conscience, conveys or does not to the healthy mind a *certain* conviction of what is morally right and wrong?

Certain writers assert, that moral responsibility is not to be decided by this principle at all, but "by the general organization of the brain;"* "that difference of conduct arises solely from difference of cerebral organization;"† and that "to talk of responsibility, and of the necessity of the punishment of one, who falling into temptation had given way to a predisposition for taking possession of whatever he could lay his hands upon, would be quite as irrational as to flog a man at the cart's tail for having become infected with scarlet fever."‡

Again, a recent writer§ in examining this question, says, "Both metaphysicians and theologians have been accustomed to speak of the moral sense or conscience as furnishing to every man an invariable rule of right and wrong, any departure from which constitutes a violation of moral or religious duty; and lawyers have taken for granted the existence of such a standard, and have made the test of the sanity or insanity of a criminal to depend upon his supposed ability or inability to distinguish right from wrong at the time of the commission of the offence." (p. 226.) "Now," he adds, further on, "it is commonly supposed that in all such cases (viz., in the discussions which every man occasionally holds with himself as to the line of conduct which he shall pursue) conscience or the moral sense should be the supreme guide, and that every other motive should be kept in subjection to its dictates; and it is surprising to us that, in spite of the continual experience of such a difficulty, both metaphysicians and theologians, to say nothing of the so-called practical men or disciples of common sense, should continue to uphold the autocratic nature of this faculty." (Ibid. p. 227.)

With the first class of objectors we care not to dispute. Dr. Davy may, unmolested by our criticism, measure for the enlightenment of our judges and juries the extent of guilt incurred by the shape of the criminal's skull-cap; and whether he adopt the *legitimate* method of Mr. Combe, "by accurate observation with the eye and hand;" or Dr.

* "Medico-legal Reflections," &c. By J Davy, M.D. P. 22. London, 1843.

† "Criminal Jurisprudence considered in Relation to Cerebral Organization." By M. B. Sampson. P. 21. 2d edit. London, 1843.

‡ Sampson, *op. cit.* p. 34.

§ "The British and Foreign Medical Review." No. 47, art. 16. July, 1847.

Skae's "principle of cubical admeasurement,"* is equally indifferent to us.

Mr. Sampson, while abrogating all moral responsibility, may, as far as we are concerned, revel unmolested in the enjoyment of the miserable quirk with which he sums up the chapters we have made our quotations from, and flatter himself, if so he can, that his theory "renders every human being alike responsible—responsible (according to the degree of his departure either in mind or body from that degree of sanity necessary to the proper discharge of his social duties) to undergo the painful but benevolent treatment which is requisite for his cure." (Ibid. p. 44.)

The opinions advocated by the writer in the "British and Foreign Medical Review," we would, on the other hand, briefly examine.

He questions, as we have seen, "the autocratic nature of the moral principle or conscience," and states, further on, "that the ethical judgments of men should be guided, not by any fixed and determined standard of right and wrong applied to actions, but by the balance of the motives which have led to the actions in question," (p. 230;) and that "it is in a great many cases difficult to decide upon the right course, simply because it is difficult to strike the balance between contending motives." (p. 229.)

Now, these objections to the "autocratic" nature of the moral principle, and to the "fixed and determined standard of its judgments," are the result of the reviewer neglecting to consider the *province* of the moral principle, which is, to decide on *moral* duties alone, not on *positive* duties, to issue moral, not positive commands.†

Moral commands arise out of the nature of a case, prior to and independent of external command. Positive duties, on the other hand, arise out of the nature of the case as influenced by external command. Our obedience to moral commands will be in proportion as we listen to the dictates of the moral principle; that to positive will bear a relation to the esteem we hold the giver of the external command in. But as our neglecting to obey positive commands is no proof that the giver of these commands (viz., the civil magistrate, a parent, &c.) does not exist, so, in like manner, does our non-obedience to moral commands form no proof against the existence of the moral principle.

While, therefore, we deny that the dictates of the moral principle ever contradict one another, we are prepared to admit that positive and moral commands do so; which shall have the preference has on several occasions been expressly determined by our Lord. "Behold," says the Evangelist, "there was a man which had his hand withered, and they asked Him, saying, Is it lawful to heal on the sabbath day? (the keeping holy of which was a positive command to the Jews.) And He said unto them, What man shall there be among you that shall have one sheep, and if it fall into a pit on the sabbath day, will he not lay hold on it and lift it

* See "The British Quarterly Journal," November, 1846; "Lancet," December, 1846; "Phrenological Journal," January and July, 1847; for the elucidation of this question. We recommend Dr. Skae's papers to the notice of those fond of clever repartee and criticism. On the merits of the question we do not presume to offer an opinion.

† Consult Bishop Butler, "The Analogy of Religion," &c. Part II. chap. 1.

out? How much, then, is a man better than a sheep? Wherefore it is lawful to do well on the sabbath days," &c. Here our Lord prefers two distinct moral commands, the reasonable care for the necessaries of life, and the doing good to others, to the positive command of keeping the Sabbath; and hence, by a parity of reason, to other ritual institutions, and, in general, moral duties to positive ones.

In opposition, then, to the objections we have considered, we assert *that the moral principle does exist in the healthy mind, and that it decides with an "autocratic" power on what is morally right and wrong.*

We confidently appeal to the personal experience of every unprejudiced reader, if there be not an inward principle, the still small voice of conscience, declaring, irrespective of outward circumstances, and in opposition often to every wish of the offender, what is moral guilt and what is not; deciding, too, though faintly, on the very principles which actuate an all-just God, "if our heart condemn us, God is greater than our heart, and knoweth all things; but if our heart condemn us not, then have we confidence toward God."

Many, however, of those who admit the existence in the healthy mind of the moral principle, deny the practicability of applying its loss as a test of the irresponsibility of a criminal, in cases in which the plea of insanity is set up. "Medical witnesses," says Dr. Guy,* "may express, in general terms, their opinion that a man is irresponsible; judges, in their charges to the jury, may insist upon the distinction; but not one of them will dare to grapple with the naked question of right and wrong; not one of them will be really guilty of such presumption."

As with the reason, its loss can alone be decided from the action of the mental powers it directs, viz., the intellectual; so also with the moral principle can its loss only be deduced from the action of the mental powers it guides, viz., the moral, or, as they are also termed, the desires and affections. The loss of the power of comprehension, the presence of a hallucination, &c., are universally and justly received in evidence that the reason is lost or impaired. And so must, by a parity of reason, total disorder of the moral powers, or perversion of one or more of the desires or affections, be received as proof that their guide, the moral principle or conscience, is lost or impaired.

But as any single absurd notion or false logic, would by no one be admitted as proof that the reason was lost; so, likewise, can no single unprincipled act or vicious conduct be admitted in evidence of the loss of the moral principle; which but leads us to our former assertion, that only those acquainted with the action of the mind in health and with its varied symptoms in disease, can decide how far any individual act is the result of the loss from disease of the consciousness of right and wrong, or the simply yielding to the tempter's power in the angry storm of passion, or in the still more sinful nurture of deadly and malevolent feeling.

For it will be observed, that as revelation declares all men to be responsible to their Creator for their acts, so, likewise, does the Law assert that all are liable to punishment, unless at the time of committing the

* "Principles of Forensic Medicine," p. 261. London, 1844.

act they were of *diseased mind*, (*i.e.* of mind altered from its normal condition), *and further, not conscious of right and wrong.*

Assuming, then, that there is a consciousness of right and wrong, and that this consciousness of right and wrong may in the diseased mind be ascertained to be present or absent, according to the condition of the moral powers, which it directs, let us examine how far in the varieties of insanity the moral principle, the consciousness of right and wrong, is lost, or otherwise, and if thereby the responsibility or irresponsibility of the criminal can be justly decided.

The different varieties of insanity may, for all practical purposes, be classed under the following heads :—

1. Idiocy or Amentia,—being the absence from birth up of all the manifestations of mind, intellectual as well as moral.

2. Imbecility, including Dr. Mayo's "Brutality,"—being a deficiency from birth, in the first instance, of the intellectual, in the other, of the moral, qualities of our nature.

3. General Insanity or Mania,—including chronic mania and dementia, and consisting in a mingled excitement and confusion of every faculty of the mind, intellectual as well as moral, proceeding to obliteration of these faculties through the stages of loss of memory; of the power of comprehension; of instinct.

4. Partial Insanity,—including moral insanity, monomania, instinctive insanity, each which variety we shall have occasion separately to describe.

5. Melancholia,—being a depression, amounting often to a suppression, of all the mental manifestations, alike of the intellectual and of the moral, which may or may not be attended with a delusion.

Idiocy we defined as an absence from birth of all the manifestations of the mind, intellectual as well as moral.

The sense of touch, taste, &c., is deficient, even to absence ; the mental powers, as attention, memory, &c., do not exist; speech is absent, or deficient,—a cry or noise, painful to listen to, being the idiot's only mode of expression; to the external sensations of pain, cold, and the like, he is more or less indifferent; the moral powers are absent,—subject to violent fits of passion, he neither cares about the result, nor is able to appreciate the consequences, of any act of violence.

The consciousness of right and wrong, as of reason, is therefore awanting; and no one would question the justice of this test in absolving him from criminal responsibility.

Imbecility and Brutality we classed together. Imbecility we defined as a deficiency from birth of the intellectual powers. The person so affected may or may not be equal to the management of his property; in every instance, his moral powers are so developed as to render him conscious of moral right and wrong. Such is Oxford. "This young man," as Dr. Mayo justly observes, "was plainly capable of appreciating his own offence in relation to the laws of the country." (p. 203.)

The legal test, "the consciousness of right and wrong," would declare such to be responsible for criminal acts. "The entire escape of Oxford," adds Dr. Mayo, "was confessedly most mischievous."

"Whether, in favour of the weak-minded, whose acts are not characterized by sufficient deliberation to entitle them to the awful punishment of death, some other punishment might not be deemed a sufficient preventive, is a question which has already met with some legislation." (p. 204.)

Brutality we defined as a deficiency from birth of the moral powers. Such instances are familiar to all; in none, however, do we conceive the moral sense to be so deficient as that the broad principles of moral right and wrong are not written on their hearts.

As such, the law regards them as responsible for criminal acts, and, we think, justly.*

To proceed, however, to those cases of "*diseased mind*," in which the consciousness of right and wrong may or may not be present, we classed under a third head, General Insanity or Mania, including chronic mania and dementia; and defined it as consisting in that mingled excitement and confusion of every faculty of the mind, intellectual as well as moral, proceeding to obliteration of these faculties, through the stages of loss of memory—of the power of comprehension—of instinct.

Here the moral principle participates in the general disorder, to obliteration; and the consciousness of right and wrong being absent, the legal test absolves such cases, and justly so, from criminal responsibility.

Under our fourth head, we classed Partial Insanity, including moral insanity, monomania, and instinctive insanity; which, as being the three varieties of mental disease which cause, as regards criminal responsibility, the greatest diversity of opinion, require separately to be considered.

*a.* Moral Insanity. This disease consists essentially in perversion of the moral powers, the intellectual powers remaining sound. It has a great tendency to proceed to intellectual disease, as will be noticed under the head of monomania.

We have seen this state supervene as the result of injury to the head; it frequently presents itself as the consequences of an attack of mania. In other instances, we have observed the exciting cause to be moral, as disappointment.

The whole inward man appears to undergo a marked change; while reasoning with perfect acuteness on every subject within his sphere of knowledge, the moral maniac is dead to the calls of social affection—of honour—or of duty, to all of which, before the supervention of this morbid state, he paid the greatest attention. "Notwithstanding," says Ray,† "the correctness of his conversation, and his plausible reasons for his singular conduct, a strict scrutiny of the actions of a patient labouring under moral insanity, if not of his words, will convince us that his notions of right and wrong are obscured and perverted, and that his own social position is viewed through a medium which gives a false colouring to its whole aspect."

The later years of the life of Frederick William of Prussia were marked by the supervention of this disease.

* In a perverted condition of the moral powers, the *result of disease*, a different opinion is to be formed,—see Moral Insanity.

† "Treatise on the Medical Jurisprudence of Insanity," p. 175. Edin. 1839.

"About a dozen years before his death," says Ray,* "his health gave way under his constant debauches in drunkenness,—he became hypochondriacal, and redoubled his usual religious austerities. He forbade his family to talk of any subject but religion, read them daily sermons, and compelled them to sing, punishing with the utmost severity any inattention to these exercises. The prince and his eldest sister soon began to attract a disproportionate share of his hostility. He obliged them to eat and drink unwholesome or nauseous articles, and would even spit in their dishes; addressing them only in the language of invective, and at times endeavouring to strike them with his crutch. About this time he attempted to strangle himself, and would have accomplished his design, had not the queen come to his assistance. His brutality towards the prince at last arrived at such a pitch, that he one morning seized him by the collar, as he entered his bedchamber, and began beating him with a cane in the cruelest manner, till obliged to desist from pure exhaustion. On another occasion, shortly after, he seized his son by the hair, and threw him on the ground, beating him till he was tired, when he dragged him to a window, apparently for the purpose of throwing him out. A servant hearing the cries of the prince, came to his assistance, and delivered him from his hands. Not satisfied with treating him in this barbarous manner, he endeavoured, though unsuccessfully, by a similar course of conduct, to make him sign an act, renouncing his claim to the succession of the Prussian throne in favour of his brother. To obtain this end, though in a different manner, he connived at the prince's attempts to escape from his tyranny, in order that he might procure from a court martial a sentence of death; and this even he was anxious to anticipate, by endeavouring to run him through the body with his sword. Not succeeding in procuring his death by judicial proceedings, he kept him in confinement, and turned all his thoughts towards converting him to Christianity. In his correspondence with the chaplain, to whom he had intrusted the charge of converting the prince, he speaks of him as one who had committed many and heinous sins against God and the king, as having a hardened heart, and being in the fangs of Satan. Even after he became satisfied with the repentance of the prince, he showed no disposition to relax the severities of his confinement. He was kept in a miserable room, deprived of all the comfort and many of the necessaries of life, denied the use of pens, ink, and paper, and allowed scarcely food enough to prevent starvation. His treatment of the princess was no less barbarous. She was also confined, and every effort used to make her situation thoroughly wretched; and though, after a few years, he relaxed his persecution of his children, the general tenour of his conduct towards his family and others evinced little improvement in his disorder till the day of his death."

The diseased perversion of the moral powers in such cases of insanity, prove (see above, p. 42) that the moral principle is diseased, and that the consciousness of right and wrong is lost. The legal test, therefore, declares such cases to be irresponsible for criminal acts; an opinion we concur in, as likewise does Dr. Mayo. "So far," he says, "as insanity involves irresponsibility, moral insanity, viewed as one of its species, or more correctly, as a train of moral symptoms occurring in its course, must also imply irresponsibility." (p. 168.)

*b.* Monomania. This disease is characterized by the presence of a delusion or series of delusions, having generally reference to the person so affected, and unattended by maniacal excitement. On subjects unconnected with his delusions, the monomaniac converses rationally, and often argues acutely regarding it.

This intellectual disorder, in very many instances, supervenes on the state of moral insanity we have just described. Such was the progress of this disease in the case of William Stalker, detailed by Dr. Lockhart Robertson, "in which morbid perversion of the moral feelings preceded the supervention of intellectual delusions, and led to the commission of murder." (p. 7.) Similar instances are related by Dr. Prichard, in his

* Op. cit., p. 124; also Lord Dover, "Life of Frederick II. King of Prussia."

excellent Treatise on Insanity. Stalker was tried at the Cumberland Lent Assizes, 1847, by Mr. Baron Alderson, and most justly acquitted, "as unconscious of right and wrong, and unfit to appreciate his position as a moral agent responsible to the laws of God and man."

There are, however, other instances of monomania, in which the disease is, to a great extent, centred in the intellectual hallucinations, in which persons, as Dr. Thurnam says, "are perfectly able to distinguish right from wrong, and in whom the moral sense is neither obliterated nor altogether perverted."* Such Dr. Mayo regards Macnaughten to be:

"In his assumption of a conspiracy against him on the part of the members of the government, he was truly insane and irresponsible; but this delusion granted, he might still be deemed capable of appreciating the fact, that to defeat a conspiracy by an act of assassination is quite contrary to the laws of this country."—P. 177.

The law, which justly acquits the morally insane, as being of diseased mind, and unconscious of right and wrong, will in like manner absolve from criminal responsibility those in whom to this disease is superadded a delusion.

Cases of monomania, on the other hand, unattended by perversion of the moral principle, the law declares to be, under all circumstances, responsible for their conduct. "Notwithstanding," say the judges, "the party committed a wrong act while labouring under the idea that he was redressing a supposed grievance or injury, or under the impression of obtaing some public or private benefit, he was liable to punishment."†

In the justice of this decision we entirely concur.

"If," says Dr. Mayo, alluding in another place to Macnaughten, "as I believe to have been the case, he was and is a monomaniac, he was not therefore *primâ facie* incapable of learning, or of teaching others, by the example of his punishment, that a strong desire of vengeance, or, if you please, of prevention of mischief, must not be gratified in defiance to the laws of his country. . . . .

"Had his friends viewed him as liable to capital punishment, in case of an outbreak against society, of which, in kind, he seems to have given them ample notice, it is reasonable to presume that some means of cure or of prevention might have been resorted to by them. . . . .

"His punishment would have afforded an invaluable example to thousands of ill regulated minds, in which a seed of insanity springs to life in a hot-bed of evil passions, or wayward dispositions, or over-stimulated imaginations . . . . The great, the good, the high-placed, who, in this country, are likely also to belong to the two first classes, are placed (*by the ignorant application of the legal test by unqualified juries and physicians*‡—*see above*, p. 39,) under the influence of circumstances over which they have no control, in direct exposure to the knife of privileged assassins; privileged indeed in two ways, for they enjoy the immunities of presumed sanity before they commit the crime, and of presumed insanity after its commission."—P. 198, *et seq.*

The correctness of these views will be further borne out when we take into consideration—

"That power which the insane are known to possess, under certain limitations, of controlling, from prudential, or other motives, their own tendencies; that power, which, under the improved management of the present age, is so often employed for their benefit, and sometimes for their cure; and which, in these instances, might be exhibited

---

* Statistics of Insanity. Foot Note, p. 48. London, 1845.

† Answers: already quoted.

‡ Or, as Dr. Mayo expresses it, "by our judicial arrangements, which leave the subtle questions of mental unsoundness at the disposition of juries."—P. 206.

in so far limiting their delusions as to render them compatible with the peace of society.'"*—P. 198.

*c.* Instinctive Insanity. This disease is described as an insane impulse to the commission of crime, occurring in a mind both intellectually and morally sound. The consciousness of right and wrong is present; the individual is stated often successfully to have resisted this striving.†

Such cases partake too much of the nature of crime to be received in exculpation of murder, and the consciousness of right and wrong being, without a question, present, the law of England rightly renders such responsible for their acts.

The cases which have led to the recognition of this disorder by systematic writers, have all been detailed by French authors — as Marc, Esquirol, Pinel, &c., and it would indeed appear as if the general absence in that country of the principles of morality and the universal indulgence of every appetite and desire, not contra-indicated by selfishness, which there occurs, together with the non-recognition and consequent neglect of His commands, who requires of his followers the cultivation of a self-denying state of mind, really produced a mental disease, consisting in a perverted state of volition, in a suspension of the free exercise of the will. But, to adopt the words of an eloquent writer,‡ "man has in the resources of his own nature the antagonist power, which, if properly used, can set at nought the evils—ay, and the so-called irresistible propensities, too—of the bodily organism. So nicely balanced, indeed, is the machine, that a grain can turn it to either side, but it is in the power of the will to cast that grain. Cast on the side of instinct, the propensity becomes passion, and the passion crime, and both are for the time insanity; for when once the intelligent will has lent its force to the blind impulses of the body, whether diseased or in health, it becomes only a question of time whether the individual is to be called insane, and placed under restraint or not. The man who recovers quickly from his madness is called a sane man, though, during the few preceding minutes or hours, he may have exhibited the flushed face, the rapid and violent language and gestures, and the unreasoning conclusions of a maniac; but, strange to say, if this be very frequent, he is excused, and considered innocent of the crimes he perpetrates, exactly be-

* So likewise writes Dr. Thurnam:—" Most of the improvements which have, of late years, taken place in the treatment of the insane, have flown from the more decided recognition of the principle of more or less power of self-control remaining in the insane; and there could hardly be anything more inconsistent with modern, and, as I believe, correct views of moral treatment, than the adoption by medical or legal authorities of the doctrine, that the plea of insanity, in *all cases* of crime, ought to be admitted in bar of punishment. There can, indeed, be no doubt that the fear of disgrace and of punishment operates strongly, and often salutarily, on many more or less partially disordered minds; and if in our courts of justice the plea in question should come to be indiscriminately admitted in all cases of partial insanity, one strong incentive to self-restraint, one important aid in the proper treatment of mental disorders, would doubtless be withdrawn, and with what amount of evil result to society I will not here presume to determine."—*Loc. cit.*

† See several instances related by Esquirol. Des Maladies Mentales, tom. ii. chap. xxi. Mémoire sur la Monomanie Homicide. Paris, 1838.

‡ Mr. Barlow: On Man's Power over himself to prevent or control Insanity. Pp. 38. London, 1843.

cause he has committed the greatest of all crimes by delivering over his god-like intellect to be the sport of that brute nature which it ought to regulate."

5. MELANCHOLIA. This disease we defined as being a depression amounting often to a suppression of all the mental manifestations, alike of the intellectual and of the moral, which may or may not be attended with a delusion.

It is evident that in this disorder the action of the moral principle is weakened, if not suspended, so that the verdict of "temporary insanity" in cases of self-destruction, (as regards which act alone, the criminal responsibility of those suffering from melancholia is ever a matter for legal decision,) is founded alike on justice and on the legal test for insanity—the loss of consciousness of right and wrong.

We have now, as far as our limits will admit of, considered the interesting question of responsibility and irresponsibility, as connected with the question of insanity, and we have seen that the legal test of the same is based on sound philosophy, as also, that *were it competently administered*, it is well adapted, while granting immunity to those by disease rendered unconscious of right and wrong, to restrain by a check they are able to appreciate—viz., the dread of punishment—the ill-regulated passions of those of the partially insane, whom disease has not deprived of this consciousness. We have not, at present, space to discuss the *nature* of the punishment best adapted to those of the latter class who infringe the laws of their country. With an extract from Dr. Mayo on this subject, we must draw our remarks to a conclusion:—

"I am far from affirming that all these unhappy persons (alluding to Macnaughten, Johnston, Martha Brixey, and the Hon. Mr. Tucket) deserved death; I only mean to suggest, that for the sake of preventive example, they required some form of punishment. To many of these criminal (so called) lunatics, the enduring confinement is in itself a dreadful punishment; but in these cases there is a sad expenditure of unfruitful suffering; for the confinement, being entirely and sedulously deprived of the character of a punishment, operates unpreventively. I waive, at present, the consideration of punishment as to its reformatory effects; though here it claims attention on behalf of the unfortunate delinquents themselves, in so far as they may be responsible agents, in a religious point of view.

". . . . Simple imprisonment, solitary confinement, transportation, as recently, in the case of Dalmas, may each find a place in such a code, whatever subsequent detention may be required for the protection of the public, when the penal period may have expired."—P. 176.

